# Diagnostic Value of Gonococcus Vaccine

**Published:** 1916-06

**Authors:** 


**Diagnostic Value of Gonococcus Vaccine.** Asch & Adler, Muench. Med. Woch., Jan. 18, advocate the use of large numbers of killed germs, 50-100 million, hypodermatically, even repeating with 125 milion once or twice if gonorrhoea is suspected in spite of an apparently negative result. Two or three days after injection, a urethral discharge occurs, containing degenerate, mainly extracellular cocci, such as are seen in cultures. The injections are repeated two or three days after the disappearance of these.

